# The intergenerational reproduction of self-direction at work: Revisiting *Class and Conformity*

**DOI:** 10.1093/sf/soaf016

**Published:** 2025-02-02

**Authors:** Kaspar Burger, Francesca Mele, Monica Kirkpatrick Johnson, Jeylan Mortimer, Xiaowen Han

**Affiliations:** Department of Education, University of Potsdam, Campus Golm, Karl-Liebknecht-Str. 24-25, 14476 Potsdam, Germany; Department of Social Sciences, University of Luxembourg, 2, place de l'Université, 4365 Esch-sur-Alzette, Luxembourg; Jacobs Center for Productive Youth Development, University of Zurich, Andreasstrasse 15, 8050 Zurich, Switzerland; Social Research Institute, Institute of Education, University College London, 55-59 Gordon Square, WC1H 0AL London, United Kingdom; Department of Education, University of Potsdam, Campus Golm, Karl-Liebknecht-Str. 24-25, 14476 Potsdam, Germany; Jacobs Center for Productive Youth Development, University of Zurich, Andreasstrasse 15, 8050 Zurich, Switzerland; Social Research Institute, Institute of Education, University College London, 55-59 Gordon Square, WC1H 0AL London, United Kingdom; Department of Sociology, Washington State University, Pullman, Washington 99164-4020, United States of America; Department of Sociology, University of Minnesota, 267 19th Ave S, Minneapolis, MN 55455, United States of America; Department of Sociology, University of Minnesota, 267 19th Ave S, Minneapolis, MN 55455, United States of America

**Keywords:** life course, social psychology, work/labor process, inequality/social stratification

## Abstract

In his path-breaking monograph, *Class and Conformity*, Melvin Kohn reasoned that parents prepare their children for the same conditions of work that they themselves experience. Kohn and his colleagues’ research focused on the influence of parental self-direction at work on parental child-rearing values and practices, as well as the self-directed values of children. The intergenerational transmission of occupational self-direction from parents to the succeeding generation of adult children, strongly implied by Kohn’s analysis, has not been empirically tested. Using two-generation longitudinal data from the Youth Development Study (*N* = 1139), we estimate a structural equation model to assess the intergenerational continuity of occupational self-direction. We find evidence supporting a key inference of Kohn’s analysis: that self-direction at work, a primary feature of jobs of higher social class standing, is transmitted across generations via self-directed psychological orientations, operationalized here as intrinsic work values. Intrinsic values also significantly predicted second-generation educational attainment, contributing further to the reproduction of socioeconomic inequality. The findings enhance understanding of the intergenerational transmission of advantage.

## Introduction

Social scientists have long been interested in the intergenerational reproduction of social class standing, with central focus on “extrinsic” work rewards, such as occupational prestige and income. However, “intrinsic” rewards—gratifications obtained from work experience itself—also affect the quality of individual lives. In the present study, we draw on Melvin Kohn’s foundational research (Kohn 1969; Kohn and Schooler 1969, 1973) that highlights a central intrinsic feature of work, what he called occupational self-direction. Whereas the more extrinsically rewarding occupational positions are typically self-directed, Kohn considered the latter a distinct occupational dimension.^1^ Using data spanning two generations and over two decades from the longitudinal Youth Development Study (YDS; 1988–2011), we interrogate the “linked lives” of parents and children (Elder, Johnson, and Crosnoe 2003) by assessing the intergenerational transmission of occupational self-direction. We highlight the socialization of intrinsic work values—a hallmark of self-directed orientation—as an intervening process, contributing to occupational self-direction in the second generation.

Kohn thought that parental values and behaviors linked to occupational self-direction played a central role in the reproduction of social inequality, as higher status and more self-directed parents socialize their children to also value self-direction and to act autonomously in the workplace (1969: 200–201). Since self-directed jobs tend to be “better” jobs, that is, more extrinsically rewarding, parents with more self-directed work give their children a leg up in the socioeconomic attainment process. Children’s self-directed orientations and behaviors equip them well for future self-directed as well as higher status work.

Kohn reasoned that children of self-directed workers would attain more self-directed work via socialization in the family (1969: 200–201), but he never tested this prediction. Although Kohn’s work has sparked considerable interest among social scientists (e.g., Hitlin and Piliavin 2004; Weininger and Lareau 2009), little attention has been directed to the intergenerational transmission of self-direction (Hout 1984). Might self-directed work be transmitted from parents to children? Could the transmission of intrinsic work values—a hallmark of self-directed orientation—help to explain such continuity? Would this process promote the social reproduction of inequality across generations? Surprisingly, despite the prominence of Kohn’s work, researchers have not explored these possibilities.

We posit four hypotheses. The first overarching rationale for this study is that occupational self-direction is transmitted from parents to children; the remaining hypotheses address processes through which this transmission occurs. The second hypothesis is that parents with more self-directed work emphasize intrinsic work values for themselves. The third is that parental intrinsic work values are transmitted to adult children; and the fourth is that the intergenerational transmission of intrinsic work values constitutes an intervening link between parents’ and children’s self-direction in work. The entire posited chain of influence elucidates intergenerational processes underlying “the perpetuation of inequality” via the transmission of occupational self-direction (Kohn, 1969, 200–201).

## Review of literature

### Intergenerational transmission of occupational self-direction

Generations of social scientists have examined processes of social class reproduction; the Wisconsin status attainment model provides the theoretical foundation for much of this research (Alexander, Eckland, and Griffin 1975; Haller and Portes 1973; Sewell, Haller, and Portes 1969). Parental education, income, and occupational prestige are reflected in offspring attainments through a chain of social psychological influences. Studies through the years document the persistence of mechanisms through which parents influence children’s life chances (Andrew and Hauser 2011; Ashby and Schoon 2010; Bozick et al. 2010; Johnson and Reynolds 2013; Reynolds and Johnson 2011; Warren, Hauser, and Sheridan 2002).^2^

Whereas the socioeconomic status or prestige of occupations, as well as their financial remuneration, are of crucial importance, they are not the only features of work that are subject to parental influence. Occupational status and income may be considered “extrinsic” rewards that people obtain for doing their jobs. However, another category of work rewards, dubbed “intrinsic,” refers to gratifications obtained from work experience itself (Johnson et al. 2007; Kraaykamp, Cemalcilar, and Tosun 2019). Intrinsic occupational features are often highlighted in studies of occupational choice and vocational development (Johnson 2001; Johnson and Monserud 2010; Johnson and Mortimer 2011; Mortimer and Lorence 1979; Weisgram, Bigler, and Liben 2010). However, the possibility that intrinsic work experiences are transmitted intergenerationally has been neglected in sociological theorizing and research. Our first hypothesis is that self-direction in work is transmitted intergenerationally. Our remaining hypotheses specify a chain of influences that may underlie this process of intergenerational reproduction.

### Parental self-direction in work and parental intrinsic work values

In his seminal early work, *Class and Conformity* (1969), Kohn posited a process of intergenerational attainment through the central medium of values and orientation. His ten-year study of a representative sample of more than three thousand American men identified significant social class differences in parental child-rearing values and practices. Middle-class parents prioritized children’s internal psychological dynamics (e.g., responsibility and intentionality); working-class parents emphasized behavioral conformity to external authority. Despite shifts in parental child-rearing values toward greater emphasis on self-direction (Alwin 1984, 1988; Wright and Wright 1976), Kohn’s findings have received repeated confirmation in multiple countries (Baker and Barg 2019; Curtner-Smith, Bennett, and O’Rear 1995; Kohn et al. 1990; Kohn and Slomczynski 1990; Tudge et al. 2000; Wright and Wright 1976).

Kohn posited that class-related differences in parental values result from a central divide in the work experiences of middle- and working-class parents, distinct “occupational conditions that are conducive to or restrictive of the exercise of self-direction in work” (Kohn 1969, 139). Kohn and his colleagues identified three components of “occupational self-direction:” (1) substantive complexity of work—work requiring initiative, thought, and independent judgment; (2) closeness of supervision—as workers cannot exercise occupational self-direction if they are closely supervised; and (3) routinization—as work, to be self-directed, must involve a variety of complexly structured tasks (Kohn 1969; Kohn and Schooler 1982; Schooler, Mulatu and Oates 2004).

Kohn initially revealed significant relationships between fathers’ self-direction at work and fathers’ child-rearing values and practices (1969, 1976), as well as mothers’ child-rearing values (1969). More important for the present study, Kohn also found that self-direction was linked to “orientations to work, society, and self” (1969, Ch. 5). According to Kohn and Schooler’s (1983) theory of “learning generalization,” as adults learn to successfully adapt to daily life pressures and circumstances encountered in the workplace, they generalize this understanding to other situations: “to the extent that environments reward initiative and independent judgment, they should foster a generalized individualistic orientation favoring self-directedness rather than conformity to external authority” (Schooler, Mulatu, and Oates 2004, 163). Thus, when success on the job depends on autonomy and initiative, workers come to value and enact the same traits in other settings. When success depends on conformity to rules and regulations established by others, they take on a conformist stance. Kohn and Schooler (1973, 1982) found wide-ranging beneficial psychological consequences of occupational self-direction, including job satisfaction, openness to new experience, internal control orientation, and intellectual flexibility (see also Miller et al. 1979; Miller, Kohn, and Schooler 1986; Mirowsky 2011). Self-direction and conformity in the workplace substantially explained social class differences across a wide range of psychological orientations, including intrinsic and extrinsic work values (Kohn and Schooler 1983).

Kohn’s thesis that occupational self-direction is key to understanding parental values and other psychological orientations is challenged by those who emphasize the primacy of education. Wright and Wright (1976), using data from the 1973 General Social Survey (GSS), found that education was a more powerful predictor of parental values than the occupational component of Kohn’s social class measure. Recently, Alwin and Tufiș (2021, 331), based on analysis of 19 GSS surveys (1986–2016), conclude that “both levels of schooling and occupational class appear to be important predictors of child-rearing values, but schooling is by far the more important factor.” Although Kohn himself found that education was more important than occupation (operationalized by the Hollingshead Index [Kohn 1969, 12]) in explaining self-directed parental values (1969, 132–133), he typically combined them in a single construct. We separate education and occupation to reveal their unique effects and to correctly specify their causal order.

Although class-related parental *child-rearing values* and practices were emphasized in *Class and Conformity*, we focus on *intrinsic work values*. Work values designate features of work that are prioritized in making initial occupational choices and in moving from job to job, as individuals seek work matching their preferences and capacities (Hitlin 2006; Kraaykamp, Cemalcilar, and Tosun 2019). They are usually (not universally, see Halaby 2003) considered bi-dimensional, highlighting key occupational rewards (Johnson et al. 2007). Extrinsic values emphasize rewards external to work itself, e.g., income, prestige, and security; intrinsic values reference rewarding experiences on the job linked to self-direction, such as autonomy, the expression of individual interests, and learning opportunities. Kohn’s research supports our conjecture that work values constitute a key link in the intergenerational transmission of occupational self-direction. In fact, Kohn (1969/1977, p. 166, Table 10-1) found that intrinsic work values were cross-sectionally related to all three dimensions of adult occupational self-direction: substantive complexity and routinization of work, and closeness of supervision. These dimensions accounted for nearly all the association between social class and parental intrinsic work values (94%), but just 57% of the social class-extrinsic values correlation (Kohn 1969/1977, p. 184, Table 10-7). Thus, our second hypothesis is that parents whose work is more self-directed will emphasize intrinsic work values for themselves.

### The transmission of intrinsic work values from parents to children

Joining a long tradition of social science theorizing and research, from Bengtson’s classic (1975) work on the transmission of religious values across multiple generations, to more recent research on the transmission of work values (Cemalcilar, Jensen, and Tosun 2019; Johnson and Mortimer 2015; Johnson, et al. 2020; Kraaykamp, Cemalcilar, and Tosun 2019) and political orientations (Jennings et al. 2009), Kohn (1983) developed a theoretical model of value transmission in the family. It included parents’ occupational position, occupational self-direction and education, values when children are older, child-rearing practices and relationships with the child, and children’s perceptions of parental values. Subsequently, Kohn and his colleagues’ empirical work (Kohn, Slomczynski, and Schoenbach 1986; Kohn and Slomczynski 1990) highlighted positive relationships among parental experiences of self-direction at work, their self-directed values, and the self-directed values of their children (children aged 13–25 and their parents evaluated the same characteristics, e.g., good manners, being neat and clean, responsible, considerate, etc.). Kohn and Slomczynski (1990, 201) concluded, “...all the links in the causal chain are strong: social structural position affects parental occupational self-direction, occupational self-direction affects parental values; parental values affect children’s values.”

Kohn’s classic studies have inspired researchers in multiple social science disciplines (Alwin 1989; Grossmann and Varnum 2011; Morgan et al. 1979; Phalet and Schönpflug 2001; Skvoretz and Kheoruenromne 1979; Spenner 1988). Scholars have attended to the socialization mechanisms through which children’s work orientations are affected by their parents’ work conditions, mainly when they are in close proximity (Kraaykamp, Cemalcilar, and Tosun 2019; Galambos and Sears 1998; Johnson and Mortimer 2015; Preves and Mortimer 2013). Parents shape their children’s work values directly through instruction as well as indirectly through their behavior (Johnson et al. 2020; Kraaykamp et al. 2019). Parents talk to their children about work broadly and occupations specifically. They sometimes bring them into their workplace. Children overhear discussions parents have about their work and also observe reactions to problems and challenges at work, as well as what stimulates satisfaction. Thus, children learn what is important to their parents about work and how jobs should be evaluated and chosen (Galambos and Sears 1998; Johnson et al. 2020). While significant similarity in work values between parents and children is observed by adolescence (Ryu and Mortimer 1996), Johnson, Mortimer, and Heckhausen’s (2020) assessment of parent–child congruence in work values from mid-adolescence to adulthood, utilizing the same data source as we do here, found the strongest associations between parent and child values when children were in their late 30s. Hence, our third hypothesis is that parental intrinsic work values are transmitted to their adult children.

### Parental self-direction, intrinsic value transmission and children’s self-direction

Kohn (1969, 200–201) expected that high levels of parental self-direction at work would lead children to prefer the same work conditions as those experienced by their parents, increasing the likelihood that they would obtain such work in adulthood, as well as occupational positions with higher socioeconomic standing. He stated (1969, 200), “..parents tend to impart to their children lessons derived from the conditions of life of their own social class …the self-directed orientation of the middle and upper classes...is well adapted to meeting the new and the problematic...it teaches children to develop their analytic and their empathic abilities. These are the essentials for handling responsibility, for initiating change rather than merely reacting to it…The family, then, functions as a mechanism for perpetuating inequality.” Kohn and Schooler (1983, 309) raised caution in assuming that “parents’ valuing self-direction or conformity to external authority necessarily implied that parents would behave appropriately to their values and even that parents’ values would be successfully transmitted to children.” But indicating his continuing interest in the possibility of intergenerational value transmission, in the same year Kohn (1983) published a theoretical model of value transmission in the family, outlined above.

Despite the plausibility of intergenerational value transmission, data constraints have prevented a rigorous empirical test of Kohn’s full “occupational linkage hypothesis” (Mortimer and Kumka 1982). With this in mind, we investigate the extent to which parents transmit intrinsic work values to their children, and whether these values lead to adult children’s self-direction in work. Specifically, our fourth hypothesis is that the intergenerational transmission of occupational self-direction from parents to adult children occurs at least partially via the transmission of intrinsic work values.

Parental experience of self-direction on the job may not be reflected in children’s job conditions when they initially enter the labor force. Due to the absence of institutional bridges from school to work for most young people in the United States (Mortimer and Krueger 2000), young labor force entrants often take considerable time to “settle into” long-lasting careers. Many hold part-time entry-level jobs as they pursue higher education and training, and then move from job to job as they seek a good fit between their values, interests and abilities and their opportunities in the labor market (Mortimer, Lam, and Lee 2015). With this in mind, we examine adult children’s self-direction at work at the ages of 37 and 38, when most will have established relatively stable occupational careers.

In summary, the present study investigates Kohn’s proposition that self-direction at work is transmitted intergenerationally, with implications for the reproduction of social inequality. Although key pieces of Kohn’s argument have been empirically supported by his and subsequent studies, the entire causal chain remains to be directly tested. To examine whether work values serve as an intervening factor, connecting self-direction in parental and adult child work experiences, we draw on two-generation survey data from the longitudinal YDS. Our multivariate structural equation model posits a chain of influences including parental education and occupational status, parental occupational self-direction, parental intrinsic values, offspring intrinsic values, and adult children’s educational attainment, occupational status, and most importantly, occupational self-direction. In analyzing this hypothesized chain of influence, we control child gender, race/ethnicity, and the structure of the family of origin, as all are known to influence adult attainments.

## Data source

The YDS (Mortimer 2003) began in 1988 with an initial sample of randomly selected ninth graders enrolled in St. Paul, Minnesota public high schools (*N* = 1139). In 1988 and 1991, questionnaires were mailed to students’ residential parents to collect information on family sociodemographic background and parental occupational characteristics. A total of 924 mothers and 649 fathers participated in the 1988 survey; 690 mothers and 440 fathers returned surveys in 1991. The present analysis merges the parent (first-generation, also called “G1” hereafter) and child (second-generation, “G2”) data to examine the long-term links between parents’ and children’s self-direction at work. It utilizes data from G1 (parents) collected in 1988 and 1991 and G2 (children) in adulthood (at age 26–27, collected in 2000, the 12th survey wave; and at age 37–38, collected in 2011, the 19th wave). Sixty-two percent of the original 1988 G2 sample members were retained in the 2011 data collection. Importantly, attrition was not related to youth socioeconomic origin or to work values. However, men, non-whites, and youth who did not have an employed parent at the outset of the study had higher risk of attrition.

## Measures

The key outcome of interest, *G2 adult self-direction at work*, is measured at age 37–38 by asking respondents to evaluate three characteristics of their primary jobs: (1) “Do you have to think of new ways of doing things or solving problems on your job?”; (2) “How much control do you have over the way you spend your time at work?”; and (3) “Overall, how much freedom do you have to make important decisions about what you do at work and how you do it?” The first item references substantive complexity and routinization, as “new ways of doing things or solving problems” signify that the work is not simple, repetitive, or highly structured. The second and third items indicate decision-making capacity. High levels of decision-making suggest that the work is not closely supervised. The responses ranged from 1 (Never/Almost no control at all/Almost none at all) to 5 (Almost always/Complete control/Complete freedom).^3^

*G1 self-direction at work* is based on the same three questions described above. The measure prioritizes G1 occupational self-direction reported in 1988 (when their focal G2 child was in 9th grade) but uses 1991 data when 1988 data were missing.

*G1 educational attainment* and *G2 educational attainment* (at age 37–38) are measured on a scale that ranges from (1) “less than high school graduation” to (9) “PhD or professional degree”. For the first generation, the data were collected in 1988 and, if not available in 1988, in 1991. Second-generation educational attainment was measured in 2011, when the respondents were 37–38 years old. The scale used to assess educational attainment was adjusted slightly to account for changes in common terminology. For instance, “community junior college degree” (for the first generation) was substituted by “associate degree” (for the second generation).

*G1* and *G2 occupational status*, capturing the prestige of primary jobs, is assessed using the Duncan occupational status score (Duncan 1961) for the first generation and the Hauser-Warren occupational status score (Hauser and Warren 1997) for the second generation. Both scales range from 1 to 100; both are rescaled here to a range of 1 to 10 to harmonize them with the other variables.

To obtain measures of G1 educational attainment, occupational status, and self-direction at the family level, we parceled each item by computing the mean for mothers and fathers (Bandalos 2002; Little et al. 2013). This parceling strategy is used in family research that focuses on the overall effects of both parents on children (Gibbings et al. 2020; Kalmijn 2024). It also offers psychometric advantages as fewer parameters are needed to represent constructs. Parcels exhibit greater reliability than items and a lower likelihood of distributional violations when individual items are nonnormally distributed (Little et al. 2013). In single parent families, this measure was obtained from the residential parent; when only one parent reported their occupational self-direction, the measure relies on that parent’s responses.

*G1* and *G2 intrinsic work values* were assessed using respondents’ ratings of the importance of six work features when seeking a job: “a chance to make my own decisions,” “a job where I have a lot of responsibility,” “a chance to learn a lot of new things,” “a job that uses my skills and abilities,” “a chance to be helpful to others or useful to society,” and “a chance to work with people rather than things” (1 = not important at all to 4 = extremely important). All reference rewards that are obtained directly from the experience of employment. For the first-generation respondents, the data were collected in 1988 and, if not available in 1988, in 1991. We parceled the G1 intrinsic work value items, computing the mean for mothers and fathers, to obtain a family-level measure. For the second-generation respondents, the value data were collected in 2000, when respondents were age 26–27, so as to precede G2 self-direction in work.

Finally, we include three control variables in the analysis reported by the second generation: G2 race (non-White = 1, white = 0) and gender (female = 1, male = 0), measured in 1988, and family structure (two parent family coded 1, other = 0), measured in 1991 (or, if not available in 1991, from the most recent adolescent report in 1990, 1989, or 1988).

## Analytic strategy

**Figure 1:**
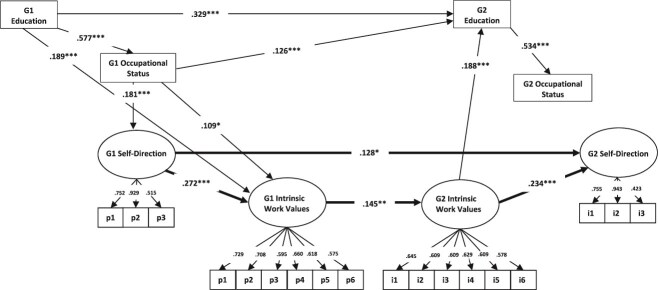
Standardized path coefficients from an initially fully recursive structural equation model (non-significant paths excluded) depicting how influence flows from first-generation variables to second-generation variables. Bold arrows correspond to the key hypotheses of the study. The model controls for second-generation race/ethnicity, gender, and the structure of the family of origin, where statistically significant. These, and residual correlations, are not depicted for the sake of readability. ^*^P < .05, ^**^P < .01, ^***^P < .001.

We estimated the theorized model in Mplus version 6.12 (Muthén and Muthén 1998–2010). Structural equation modeling is ideally suited for this study. It minimizes measurement error through the estimation of latent constructs with multiple indicators. It allows for modeling multiple endogenous variables as well as both direct and indirect effects through intervening variables. As illustrated in figure 1, influence flows in our model from first-generation (parent) characteristics to second-generation (adult children’s) characteristics (assessed at age 26–27 and 37–38). The bold arrows represent the key paths of interest. We estimate (1) a direct effect of parental self-direction on adult children’s self-direction, (2) a direct effect of parents’ self-direction at work on their own intrinsic work values, and (3) a direct effect of parental intrinsic work values on adult children’s intrinsic work values. Moreover, we evaluate (4) whether the intergenerational transmission of intrinsic work values constitutes an intervening link between parents’ and children’s occupational self-direction by investigating indirect effects. We include indicators of socioeconomic status for both generations—educational attainment and occupational status—and estimate indirect effects of the parental socioeconomic status indicators on offspring socioeconomic status indicators via parental and child self-directed orientation. This model specification allows us to assess the extent to which the posited chain of influence is implicated in the intergenerational transmission of inequality. Importantly, in addition to estimating the paths of key theoretical interest (bold arrows in fig. 1), we estimate a fully recursive model to identify those effects net of all other influences controlled in the model. Following recommendations and for parsimony, in the final model we trim nonsignificant paths while keeping residual correlations even if they were not significant (Kline 2016).

In both generations, educational attainment, occupational status, and self-direction at work are measured at the same time, but figure 1 specifies that educational attainment and occupational status precede self-direction. In a passage quoted earlier justifying their causal model, Kohn and Slomczynski (1990, 201) allege that “social structural position affects occupational self-direction.” As we pointed out above, Kohn considered variation in occupational self-direction as the reason why individuals in higher and lower social class positions vary in their orientations to self and society. Kohn’s assessment of the reduction in the magnitude of the association between social class and self-directed orientations when occupational self-direction is controlled (1969/1977) reflects this conceptualization. Thus, Kohn’s work provides justification for our specification of self-direction at work as intervening between socioeconomic status indicators (educational and occupational attainment) and intrinsic work values. We also separate educational attainment and occupational status to appropriately specify their causal order from a life course perspective.

Figure 1 specifies the relationship between self-direction at work and intrinsic values differently in the first and second generations. Although Kohn and Schooler (1983) emphasized the impacts of occupational self-direction on self-directed ways of thinking among adults, they recognized that the relationships between work and personality across time are reciprocal, indicating “socialization” as well as “selection” processes. For example, they point out that “both ideational flexibility and a self-directed orientation lead, in time, to more responsible jobs that allow greater latitude for occupational self-direction” (1982, p. 1282). Whereas socialization effects (the effects of work conditions on personality) were found to be generally contemporaneous, selection effects (effects of personality on work conditions) were more often lagged, implying that “job conditions are not readily modified to suit the needs or capacities of the individual worker.” The authors conclude, “We now have strong evidence that job conditions actually do affect personality, and also that personality affects job conditions” (Kohn and Schooler 1982, p. 1281). Clearly, Kohn and Schooler’s emphasis in studying adults was on the socialization process—how job conditions influence ways of thinking. But the mechanism underlying hypothesized intergenerational continuity in self-direction emphasized selection—young people whose self-directed orientations were compatible with self-directed work would be drawn to such jobs, or perhaps selected (by others) for them. Hence, we specify the contemporaneous relationship in G1 between parental self-direction at work and parental intrinsic values as a socialization process; in G2, we model the relationship between lagged intrinsic values and self-direction as demonstrating selection.

Missing data represent a challenge in most longitudinal studies, potentially limiting the generalizability of findings. In the present case, we had 11.1% missing values on average across items and waves.^4^ To adjust parameter estimation to the presence of missing data, we use full information maximum likelihood estimation (FIML), an efficient technique to estimate population parameters when data sources include missing values (Dong and Peng 2013). FIML estimation draws on all available data. It relies on the assumption that missing values of a variable are conditionally dependent on other observed variables; incorporating vectors of partly complete data in the likelihood function implies probable values for the missing data in the estimation process (Enders and Bandalos 2001). FIML is widely used because it generates more consistent estimates and reduces the risk of biased parameter estimation relative to traditional techniques of handling missing values such as pairwise or listwise deletion (Little et al. 2014).^5^

We assess model fit using three widely used indices: the comparative fit index (CFI), the Tucker–Lewis index (TLI), and the root mean square error of approximation (RMSEA), which are sensitive to different types of model misspecification (Hu and Bentler 1998). Model fit is commonly considered acceptable when CFI > .90, TLI > .90, and RMSEA < .08 (Kline 2016; McDonald and Ho 2002).

## Findings

### Descriptive statistics

Table 1 reports descriptive statistics for all variables. First- and second-generation respondents exhibited similar levels of occupational self-direction and occupational status, but there was somewhat greater variation in occupational self-direction among first-generation respondents. Indicating educational upgrading across generations, graduation from high school (coded 2) was the modal level of attainment for the first generation; for adult children it was some college (coded 6). Intrinsic values were quite comparable between parents and their adult children.

**Table 1:** Descriptive statistics.

Measures	Measured in	Mean	SD	Min.	Max.
First generation, in adulthood					
Mother’s highest educational attainment^a^	1988/1991	2.00^(mode)^	—	1	8
Mother’s occupational status^b^	1988	4.04	1.36	1.47	8.11
Father’s highest educational attainment^a^	1988/1991	2.00^(mode)^	—	1	8
Father’s occupational status^b^	1988	4.19	1.46	1.58	8.11
Mother’s self-direction	1988^c^				
Control over work time^d^		3.47	1.28	1	5
Freedom to make decisions^d^		3.43	1.17	1	5
Think of new ways of doing things^d^		3.58	1.05	1	5
Father’s self-direction	1988^c^				
Control over work time^d^		3.59	1.19	1	5
Freedom to make decisions^d^		3.59	1.10	1	5
Think of new ways of doing things^d^		3.82	1.08	1	5
Mother’s intrinsic work values	1988^c^				
Importance of getting a chance to make own decisions		3.01	0.77	1	4
Importance of having a job with a lot of responsibility		2.63	0.85	1	4
Importance of getting a chance to learn new things		3.14	0.77	1	4
Importance of using skills and abilities		3.29	0.70	1	4
Importance of being helpful to others or useful to society		3.12	0.79	1	4
Importance of being able to work with people rather than things		2.89	0.92	1	4
Father’s intrinsic work values	1988^c^				
Importance of getting a chance to make own decisions		3.03	0.77	1	4
Importance of having a job with a lot of responsibility		2.54	0.87	1	4
Importance of getting a chance to learn new things		2.98	0.82	1	4
Importance of using skills and abilities		3.25	0.71	1	4
Importance of being helpful to others or useful to society		2.88	0.86	1	4
Importance of being able to work with people rather than things		2.50	0.95	1	4
Second generation, in adolescence					
Gender (1 = female)	1988	0.52	—	0	1
Race (1 = non-White)	1988	0.33	—	0	1
Family structure (1 = two-parent family)	1991^e^	0.57	—	0	1
Second generation, in adulthood					
Intrinsic work values	2000				
Importance of getting a chance to make own decisions		3.02	0.72	1	4
Importance of having a job with a lot of responsibility		2.60	0.78	1	4
Importance of getting a chance to learn new things		3.09	0.77	1	4
Importance of using skills and abilities		3.30	0.72	1	4
Importance of being helpful to others or useful to society		2.82	0.83	1	4
Importance of being able to work with people rather than things		2.67	0.93	1	4
Self-direction	2011				
Control over work time^d^		3.55	1.05	1	5
Freedom to make decisions^d^		3.47	0.98	1	5
Think of new ways of doing things^d^		4.01	0.97	1	5
Highest educational attainment^a^		6.00^(mode)^	—	1	9
Occupational status^b^		4.22	1.39	1.57	8.05

NOTE*—*

^a^The mean values of educational attainment were 3.45 for fathers, 3.17 for mothers, and 5.53 for second-generation respondents. Because educational attainment is measured using an ordinal variable, the mode is reported rather than the mean which would not be readily interpretable.

^b^The item was rescaled from the original range 1–100 to 1–10 to harmonize it with other variables.

^c^When data were missing in the 1988 wave, data from the 1991 wave were substituted.

^d^The original item was reverse coded.

^e^When data were missing in 1991, the most recent data from 1990, 1989, or 1988 were used.

### Results from the structural equation model

Figure 1 depicts statistically significant standardized path coefficients from the structural equation model. From left to right, it represents first-generation educational attainment and occupational status, occupational self-direction, and intrinsic work values; second-generation intrinsic work values (at age 26–27), educational attainment, occupational status and occupational self-direction (at age 37–38). In the estimation of offspring characteristics, the analysis includes controls for second-generation race/ethnicity and gender, and family of origin structure (not shown). Table 2 reports all regression coefficients including these paths as well as all indirect effects, the latent factor loadings (ranging from 0.515 to 0.943), residual correlations, and model fit statistics. Considering the path coefficients, most effect sizes are not large. It is important to note, however, that they are net of all other effects estimated in the model. Moreover, G1 and G2 variables are separated by long time periods, which make large effect sizes unlikely and renders even weak effects noteworthy.

**Table 2:** Standardized path coefficients, latent factor loadings, residual correlations, and model fit statistics.

Structural model														
	G1 occupational status		G1 self-direction		G1 intrinsic values		G2 intrinsic values		G2 education		G2 occupational status		G2 self-direction	
	Direct	Indirect	Direct	Indirect	Direct	Indirect	Direct	Indirect	Direct	Indirect	Direct	Indirect	Direct	Indirect
G1 education	0.577^***^	—	—	0.105^***^	0.189^***^	0.091^**^	—	0.041^**^	0.329^***^	0.081^**^	—	0.219^***^	—	0.023^**^
G1 occupational status			0.181^***^	—	0.109^*^	0.049^***^	—	0.023^*^	0.126^**^	0.004^*^	—	0.070^**^	—	0.029^**^
G1 self-direction					0.272^***^	—	—	0.039^**^	—	0.007^*^	—	0.004^*^	0.128^*^	0.009^*^
G1 intrinsic values							0.145^**^	—	—	0.027^*^	—	0.015^*^	—	0.034^*^
G2 intrinsic values									0.188^***^	—	—	0.100^***^	0.234^***^	—
G2 education											0.534^***^	—	—	—
G2 occupational status													—	—
G2 controls														
Non-White							—	—	—	—	−0.107^*^	—	−0.121^*^	—
Female							0.185^***^	—	0.081^*^	0.035^**^	−0.087^*^	0.062^**^	—	0.043^**^
Two parent family							0.138^**^	—	0.114^**^	0.026^**^	—	0.074^***^	—	0.032^**^
**Latent factor loadings**														
							**G1 self-direction**		**G2 self-direction**		**G1 intrinsic values**		**G2 intrinsic values**	
Control over work time^(p)^							0.752^***^		0.755^***^					
Freedom to make decisions^(p)^							0.929^***^		0.943^***^					
Think of new ways of doing things^(p)^							0.515^***^		0.423^***^					
Importance of getting a chance to make own decisions^(p)^											0.729^***^		0.645^***^	
Importance of having a job with a lot of responsibility^(p)^											0.708^***^		0.609^***^	
Importance of getting a chance to learn new things^(p)^											0.595^***^		0.609^***^	
Importance of using skills and abilities^(p)^											0.660^***^		0.629^***^	
Importance of being helpful to others or useful to society^(p)^											0.618^***^		0.609^***^	
Importance of being able to work with people rather than things^(p)^											0.575^***^		0.578^***^	
**Residual correlation between**		**and**												
G2 occupational status		G2 self-direction					0.057							
Female		Non-White					0.012							
Female		Two parent family					−0.016							
Non-White		Two parent family					−0.144^***^							
**Model fit statistics**														
*N*						963								
χ^2^ (df)						741.627 (259)								
CFI						0.904								
TLI						0.891								
RMSEA (90% CI)						0.044 (0.040, 0.048)								

NOTE*—*Standardized path coefficients from an initially fully recursive model with non-significant paths excluded. ^(p)^ Parcel created for G1 by averaging the correspondent items collected from mothers and fathers. Negatively worded items were reverse coded. *df* = degrees of freedom. CFI = comparative fit index. TLI = Tucker Lewis index. RMSEA = root mean square error of approximation. CI = confidence interval.

^*^ *P* < .05

^**^ *P* < .01

^***^ *P* < .001

We now address our central hypotheses. First, we hypothesized that occupational self-direction is transmitted from parents to children. In line with this hypothesis, we find a small but significant total association between G1 and G2 self-direction (*β =* .128 + .009 = .137, *P* < .01; see also Appendix Table A1). Importantly, figure 1 documents a chain of influences that promotes intergenerational continuity in occupational self-direction despite considerable historical change over the course of the observation period. That is, G1 occupational status significantly predicts G1 self-direction, which, in turn, has a positive effect on G1 intrinsic values. This latter finding lends support to our second hypothesis that parents with more self-directed work emphasize intrinsic work values for themselves. Moreover, consistent with our third hypothesis, G1 intrinsic values significantly influence G2 intrinsic values measured in the mid-twenties. Importantly, our fourth hypothesis posited that the intergenerational transmission of intrinsic work values constitutes an intervening link between parents’ and children’s self-direction in work. Our findings provide support for this hypothesis, as the indirect effect of G1 self-direction on G2 self-direction is statistically significant (*β* = .009, *P* < .05; shown in Table 2). That is, G1 self-direction is significantly associated with G2 self-direction through the hypothesized intervening variables, G1 and G2 intrinsic values. This pattern supports the argument that the intergenerational transmission of occupational conditions occurs, at least partially, through the intergenerational transmission of self-directed values.

It is possible that parents’ self-direction in work provides access to self-directed jobs through parental contacts and other mechanisms, independent of parents’ and children’s values, but such alternative pathways are beyond the scope of the present study. Here, we find a direct effect of G1 self-direction on G2 self-direction, as well as a significant chain of pathways from G1 self-direction to G2 self-direction via intrinsic values. Taken together, these findings call into question sociologists’ near exclusive concern with socioeconomic status in studying intergenerational attainment and the reproduction of social inequality.

It is worth noting that occupational status predicted occupational self-direction in the first generation, whereas educational attainment did not (directly). That is, parents with higher occupational standing tend to exhibit higher levels of self-direction. That higher self-direction, as well as higher educational attainment and occupational status, promote parents’ intrinsic work values, which they pass on to their children. Second-generation work values promote higher educational attainment, which ultimately results in higher occupational status. The path from G2 intrinsic values to G2 educational attainment, and the subsequent path from educational attainment to occupational status, confirm Kohn’s prediction that self-directed values of adult children would promote their socioeconomic attainment. That is, their self-directed orientation would equip them well for higher status work, which tends to be more self-directed work. Note, however, that we only find evidence of an indirect effect of intrinsic work values on occupational status through educational attainment (*β* = .100, *P* < .001). The nonsignificant paths from G2 education and occupational status to G2 self-direction are partially attributable to the fact that G2 intrinsic values, a significant antecedent of G2 self-direction, is controlled; when the effect of G2 intrinsic values on G2 self-direction is dropped, the effect of G2 education on G2 self-direction increases to a statistically significant *β* = .230 (*P* < .05) though the effect of G2 occupational status on G2 self-direction remains non-significant (*β* = .083, *P* > .10).

Furthermore, we find a sizable *direct* effect of G1 educational attainment on G2 educational attainment, and a *direct* effect of G1 occupational status on G2 educational attainment, which in turn significantly predicts G2 occupational status. These direct effects are in no way contradictory to Kohn’s central thesis, as he never postulated that occupational self-direction and associated self-directed orientations were *the only* mechanisms linking the socio-economic status of parents and children. Parents’ high occupational status provides access to many advantages (educational investment, social and cultural capital, etc.) that facilitate offspring attainments. The indirect effect of G1 occupational status on G2 self-direction is also statistically significant (*β* = .029, *P* < .01; Table 2), as is the indirect effect of G1 educational attainment on G2 self-direction (*β* = .023, *P* < .01; Table 2).

Taken together, our findings document direct and indirect links between first- and second-generation educational and occupational attainments and occupational self-direction; they also indicate that intrinsic work values are implicated in the reproduction of social inequality.^6^ Finally, they illustrate a process of socialization through which occupational self-direction is perpetuated intergenerationally.

## Discussion

This research is rare in its consideration of the intergenerational transmission of a dimension of work other than occupational status or income. Self-direction on the job is not incorporated in the classic status attainment paradigm guiding the sociological study of the persistence of social inequality across generations. Still, study of the familial transmission of work-related phenomena to offspring, other than the vertical status or prestige dimension, is not without precedent. Most prominently, sociologists and vocational psychologists have examined the inheritance of particular occupations (Lentz and Laband 1989; Mortimer 1974), or categories of occupations, such as self-employed entrepreneurs (Aldrich and Kim 2007), teachers (Gubler, Biemann, and Herzog 2017; Jacinto and Gershenson 2021), or the “liberal professions” (Aina and Nicoletti 2018). Jonsson et al. (2009) find substantial evidence across four countries that occupational inheritance occurs within micro-classes of functionally similar occupations rooted in the institutional structures of economic production (see also Weeden and Grusky 2005). Most recently, York et al. (2025) examined the intergenerational transmission of ten gradational features of work derived from O’Net ratings (National Center for O’Net Development 2020) linked to the full range of detailed occupational categories; some of these imply self-directed thought and action (e.g., problem solving, responsibility/leadership, judgment/interpretation). Our study breaks new ground in investigating the intergenerational transmission of a self-direction construct based on parents’ and children’s own reports of key occupational experiences.

The sizable direct path from first-generation educational attainment to second-generation educational attainment, and the indirect path from first-generation occupational status to second-generation occupational status through second-generation educational attainment, are fully consistent with decades of research on status attainment. But the key focus of the present community-based panel study, extending across 23 years, is Kohn’s (1969/1977) proposition that self-direction in work is also transmitted across generations, mediated by the self-directed orientation of parents and children. Kohn found that high parental socioeconomic standing was associated with occupational self-direction, which, in turn, was linked to parental self-directed orientations that were passed on to children. However, he and his colleagues were not able to investigate Kohn’s complete hypothetical sequence through to the final link in the causal chain, from adult offspring self-directed orientation to offspring occupational self-direction. Our assessment of the entire linkage model provides evidence that self-direction, an important intrinsic quality of work, is maintained across generations through a socialization process that features intrinsic work values. Additional dynamics, beyond intrinsic values and other self-directed orientations, could well contribute to such intergenerational transmission of occupational conditions, including informal contacts that provide access to self-directed jobs as well as the possibility that parents and offspring face similar constraints on occupational placement (e.g., aspects of a shared local labor market). Evidence for the intergenerational transmission of occupational self-direction via self-directed orientation is theoretically important since it suggests processes of work-related socialization in the family that extend beyond income and prestige.

Although many other mechanisms could contribute to the intergenerational transmission of socioeconomic status, including psychological, social, and genetic processes (Branje et al. 2020), Kohn hypothesized that offspring self-directed orientation would equip adult children well for higher status occupations, and hence, higher adult socioeconomic position. We find evidence, consistent with Kohn’s formulation, that parental self-directed orientation (here represented by intrinsic values) influences adult children’s self-directed orientation, which, in turn, has a positive effect on offspring educational attainment, an important driver of occupational status attainment. Adult children’s intrinsic work values also predict their occupational self-direction.

Change in value priorities surrounding work across the historical period studied might contribute to the absence of a link between second-generation occupational status and occupational self-direction. Some research suggests that extrinsic values (e.g., status and money) have become more important to recent cohorts compared to prior ones (Twenge, Campbell, Hoffman, and Lance 2010), especially among individuals who have moved beyond initial labor force entry (Jin and Rounds 2012).

The predominant interest in the social reproduction of the extrinsic rewards of work—occupational status and income—is fully warranted, given their consequences for numerous dimensions of life chances, including standard of living, economic security, the capacity to accumulate wealth, and both physical and mental health. However, Kohn and Schooler’s research program also detailed extensive psychological ramifications of occupational self-direction, including lessening self-deprecation, fatalism and anxiety, and enhancing self-confidence, social trust, and moral standards (Kohn and Schooler 1982). The widespread psychological benefits of self-directed work have been confirmed in more recent studies (Clausen et al. 2022; Le, Donnellan, and Conger 2014; Ross 2000; Wu, Griffin, and Parker 2015). The pervasive implications of occupational self-direction for individual well-being fully warrant further attempts to understand the sources of this highly consequential work-related experience within the family and elsewhere.

While the current study indicates that the transmission of self-directed orientation plays a role in the intergenerational reproduction of inequality, we were not able to assess the exact “occupational linkage hypothesis” posited by Kohn and Schooler. The YDS collected no measures of G1 *child-rearing* values and practices, the key factors of interest in *Class and Conformity*. Nor do we have access to indicators of intellectual flexibility, also linked to occupational self-direction and of great interest to Kohn and his colleagues. Intellectual flexibility and associated problem-solving capacity might similarly intervene in the intergenerational transmission of occupational self-direction and socioeconomic status. Other self-directed psychological orientations, such as self-efficacy or openness to new experience in parent and child generations, might also play a mediating role and merit future attention.

Jonsson et al. (2009) lay out plausible mechanisms of intergenerational occupational attainment deserving further study, including the transference of human capital, encompassing work-related capacities, and cultural capital, referencing values and attitudes. Kohn and Schooler’s formulation captures both forms of capital. Children of more self-directed parents may develop the capacity to do self-directed work by becoming self-directed themselves if their parents encourage them to be curious, responsible, and independent. Their parents may provide toys, games, and spend time with them in ways that reinforce self-directed action. Children may also visit parents’ workplaces, hear their parents talk shop with one another, or receive explicit messages from their parents about the desirability of self-direction at work. Through these experiences, children may internalize parents’ values regarding the importance of self-direction on the job. Youth’s capacity to do self-directed work and their evaluation of it would likely influence their career decisions and, following employment, attempts to mold their jobs accordingly. Future research, including both survey-based and qualitative work, should further address the processes that promote the intergenerational transmission of self-directed work.

Despite the contribution of the present study to understanding the psychological dynamics underlying the attainments of occupational self-direction and socioeconomic status, it does have notable limitations. As in most observational studies, the path coefficients of our model cannot be interpreted as unbiased causal estimates, given potential omitted variables. Unobserved variables might be confounding factors; alternatively, they could moderate or mediate the effects reported here.

Also, specific features of the YDS must be considered. Because the YDS is a community-based study originating in the upper Midwest, it cannot be assumed that the same processes would characterize more representative samples of families in the nation at large. Moreover, because the YDS panel was drawn in the Fall of 1987, it mirrors the composition of the school population at that time. Since then, the St. Paul community has become much more racially and ethnically heterogeneous. Although non-whites in the YDS panel had lower levels of occupational self-direction, the small non-white component of the sample is highly diverse (including Asian Americans, African Americans, Native Americans, and Hispanic Americans). Thus, this research cannot speak to racially/ethnically specific processes of intergenerational occupational reproduction. The relatively small YDS panel also precludes assessment of other bases of heterogeneity, for example, differences in processes of intergenerational reproduction of self-directed work across father–son, father–daughter, mother–son, and mother–daughter dyads. We are also limited by the YDS questionnaire items. Whereas Kohn (1969) relied on detailed descriptions of work tasks, provided in face-to-face interviews and coded by occupational analysts, our measurement of self-direction is admittedly more subjective, based on respondents’ reports of problem solving, control over the way they spend time at work, and their freedom to make decisions.

Finally, the second-generation panel, mostly born in 1973–74, cannot be considered typical of earlier or more recent cohorts. It made the transition from education to work in the highly favorable economic climate of the mid-to-late 1990s. Contemporary cohorts are facing more precarious and uncertain labor market conditions that could possibly disrupt processes of work-related socialization in the family, severing connections between parents’ and adult children’s occupational experiences and preferences.

These data limitations suggest promising avenues for further investigation. Research conducted with larger, more representative samples would address the generalizability of the findings. Although we have little reason to expect that the intergenerational transmission of self-directed work would differ fundamentally for racial/ethnic groups or for more recent cohorts, this remains to be seen.

## Conclusion

The present study provides evidence that occupational self-direction, a key intrinsic dimension of work, is transmitted from parent to child. Though not all steps in Kohn and Schooler’s stipulated causal chain could be tapped, our findings are consistent with the “occupational linkage hypothesis” that predicts continuity of self-directed work experience across generations. Parental occupational status is found to be associated with parental self-direction at work, which, in turn, is linked to a key parental self-directed orientation, intrinsic occupational values. But most importantly, the present study addresses major gaps in the empirical assessment of the Kohn/Schooler model, the hypothesized links among the parent’s self-directed orientation, the adult child’s self-directed orientation, and the self-directed work experience of the adult offspring. We find evidence that the transmission of an intrinsic orientation toward work from parent to child fosters both educational attainment, a strong predictor of occupational status attainment, and self-direction in the next generation. The results, taken in tandem, indicate the merit of extending sociological models of intergenerational attainment to include both the self-directedness of parental work and self-directed orientations in the parental and offspring generations.

## Supplementary Material

sf-jan-24-028-File003_soaf016

## Data Availability

YDS data are publicly available at the Inter-University Consortium for Political and Social Research archive at the University of Michigan (ICPSR 24881).

## Footnotes

1 For example, truck drivers have relatively low socioeconomic status, but some have considerable autonomy, while others are required by their employers to follow clearly designated routes and schedules.

2 Research also documents material and institutional mechanisms of intergenerational transmission of status (e.g., Calarco 2018; Chetty et al. 2014; Kornrich and Furstenberg 2013; McLanahan and Percheski 2008); such structural pathways and family socialization processes are not mutually exclusive.

3 These self-direction indicators are different from Kohn’s (1969), which addressed substantive complexity of work with data, people and things; closeness of supervision; and the organization of work, e.g., what it takes to do a complete job.

4 G1 occupational status exhibited much missingness (23.8%). During the data collection period (1988 and 1991), many mothers were out of the labor force and did not answer questions regarding occupation. If they were also single parents, we would have no data from nonresidential fathers. We would also have no data from residential fathers if they did not participate in the data collection.

5 As a robustness test, we replicated our analysis using multiple imputation (MI) instead of FIML. For each missing value, we generated 20 imputed values, using the default covariance approach with Markov Chain Monte Carlo (MCMC) iterations. The replication essentially confirmed all results. Other than minor differences in the coefficients across the two strategies, two paths were significant in the FIML approach that were not significant in the MI approach, notably the effect of gender on G2 occupational status and the effect of G1 occupational status on G1 intrinsic work values. Neither of these paths are theoretically central to the study’s purpose.

6 Sensitivity analyses, replacing intrinsic work values in both generations with extrinsic values, showed that intergenerational linkages in extrinsic work values do not mediate the transmission of occupational self-direction.

## About the authors

**Kaspar Burger** is an Associate Professor at the University of Potsdam and an Honorary Associate Professor at University College London. His research interests include social inequalities, education, and the life course. His scholarly work has appeared in journals such as *Advances in Life Course Research*, *Developmental Psychology*, *European Sociological Review*, and *Social Science Research*.

**Francesca Mele** is a scientific collaborator at the University of Potsdam and a PhD candidate in Sociology at the University of Zurich. Her research interests include the life course, social inequality, youth educational trajectories, and competence development. Her recent work has appeared in the *Journal of Youth and Adolescence*.

**Monica Kirkpatrick Johnson** is Regents Professor of Sociology at Washington State University. Her research examines interrelated work, family, and education experiences across the life course, with particular focus on well-being and achievement in adolescence and the transition to adulthood. Her recent work appears in *Journal of Marriage and Family*, *Social Psychology Quarterly*, *Work and Occupations*, and *Social Forces.*

**Jeylan Mortimer** is Professor Emeritus of Sociology at the University of Minnesota. Her research interests include the impacts of work experiences on health, intergenerational attainment processes, and attitude development through the life course. Her recent work appears in *Social Forces, Journal of Research on Adolescence*, *Longitudinal and Life Course Studies*, *Discover Social Science and Heath, Developmental Psychology*, *Journal of Youth and Adolescence*, *Work and Occupations*, and *Social Science Research.*

**Xiaowen Han** is a PhD candidate in Sociology at the University of Minnesota. Her research focuses on social stratification, work and occupations, and health disparities using a life course approach. Her recent work on labor market impacts on health has been published in *Journal of Health and Social Behavior*, *Longitudinal and Life Course Studies*, and *Social Science & Medicine*.
