# Supplementary material for: The intergenerational reproduction of self-direction at work: Revisiting Class and Conformity
Source: Soc Forces. 2025 Feb 2;104(1):320–40. doi: 10.1093/sf/soaf016 (PMC12255866; doi:10.1093/sf/soaf016)
Supplement: sf-jan-24-028-File003_soaf016 [file sf-jan-24-028-file003_soaf016.pdf]

Appendix

TABLE A1. TOTAL EFFECTS

	G1 Occupational Status	G1 Self Direction	G1 Intrinsic Values	G2 Intrinsic Values	G2 Education	G2 Occupational Status	G2 Self Direction
G1 Education	.577***	.105***	.280***	.041**	.410***	.219***	.023**
G1 Occupational Status		.181***	.158***	.023*	.131**	.070**	.029**
G1 Self Direction			.272***	.039**	.007*	.004*	.137**
G1 Intrinsic Values				.145**	.027*	.015*	.034*
G2 Intrinsic Values					.188***	.100***	.234***
G2 Education						.534***	--
G2 Occupational Status							--
<u>G2 Controls</u>							
Nonwhite				--	--	-.107*	-.121*
Female				.185***	.116**	-.025	.043**
Two Parent Family				.138**	.139***	.074***	.032**

NOTE. — Standardized coefficients.

* $p < .05$, ** $p < .01$, *** $p < .001$.
